# Study of the Sound Absorption Properties of 3D-Printed Open-Porous ABS Material Structures

**DOI:** 10.3390/polym12051062

**Published:** 2020-05-06

**Authors:** Martin Vasina, Katarina Monkova, Peter Pavol Monka, Drazan Kozak, Jozef Tkac

**Affiliations:** 1 Faculty of Mechanical Engineering, VŠB-Technical University of Ostrava, 17. listopadu 15/2172, 708 33 Ostrava-Poruba, Czech Republic; 2 Faculty of Manufacturing Technologies, Technical University in Kosice, 080 01 Presov, Slovakia; peter.pavol.monka@tuke.sk (P.P.M.); jozef.tkac@tuke.sk (J.T.); 3 Faculty of Technology, Tomas Bata University in Zlin, Nam. T.G. Masaryka 275, 760 01 Zlin, Czech Republic; 4 Mechanical Engineering Faculty in Slavonski Brod, Josip Juraj Strossmayer University of Osijek, Trg Ivane Brlic-Mazuranic 2, HR-35000 Slavonski Brod, Croatia; dkozak@sfsb.hr

**Keywords:** Acrylonitrile Butadiene Styrene, sound absorption, 3D printing technology, frequency, thickness, air gap

## Abstract

Noise pollution is a negative factor that affects our environment. It is, therefore, necessary to take appropriate measures to minimize it. This article deals with the sound absorption properties of open-porous Acrylonitrile Butadiene Styrene (ABS) material structures that were produced using 3D printing technology. The material’s ability to damp sound was evaluated based on the normal incidence sound absorption coefficient and the noise reduction coefficient, which were experimentally measured by the transfer function method using an acoustic impedance tube. The different factors that affect the sound absorption behavior of the studied ABS specimens are presented in this work. In this study, it was discovered that the sound absorption properties of the tested ABS samples are significantly influenced by many factors, namely by the type of 3D-printed, open-porous material structure, the excitation frequency, the sample thickness, and the air gap size behind the sound-absorbing materials inside the acoustic impedance tube.

## 1. Introduction

Noise pollution currently has a major impact on the environment, human health, and the economy. In general, noise control includes active and passive management [1]. An active noise control (ANC) system cancels undesirable sound based on its superposition attitude [2]. An ANC system features electroacoustic equipment, which is built based on destructive intervention by creating an antinoise of equal amplitude and an opposite phase to the unwanted sound [3]. ANC systems that use no noise-absorbing materials [4] are effective for low-frequency noise. In contrast, passive noise control (PNC) systems that use sound-absorbing materials are relatively large, expensive, and ineffective with low frequencies [2,5]. Regarding low frequencies, this is because the thickness of the absorbers is insufficient compared to the acoustic wavelength [4]. For this reason, PNC systems are effective only for high-frequency noise [6]. Porous materials (e.g., foam or glass wool) are usually applied to reduce undue noise [7]. New types of suitable sound-absorbing materials are continuously being developed. Compared to conventional manufacturing technologies, it is possible to produce sound-absorbing materials with advanced open-pore structures using a 3D printing technique.

Fakrul et al. [8] studied the use of a 3D-printed lattice structure as a sound absorber. Their paper investigates the sound absorption characteristics of a lattice structure produced by additive manufacturing to improve the acoustic characteristics that cannot be fulfilled by a typical sound absorber. The study confirmed that lattice structures can absorb sound mostly above 1 kHz. Sim et al. [9] were involved in the preparation of nanocomposite films of polycarbonate/polyacrylonitrile-butadiene-styrene (PC/ABS), and investigated not only the rheological and mechanical properties of this material, but also its ability to absorb sound. The PC/ABS thin films were made by a simple solvent casting method using phenylene modified-mesoporous silica materials as additives and dichloromethane as a solvent. The tensile properties of these materials were experimentally tested at ambient temperature according to ASTM D638. The authors discovered that depending on the production process, the PC/ABS nanocomposites containing mesoporous materials could also be used as sound insulation materials. Wang et al. [10] investigated the production of a lattice structure by an additive method, the geometry of which was optimized through graded mesostructures. The design of the geometry of the global cell structure, as well as the microstructure distribution in the material and its scale, reflect load bearing. Cao et al. [11] studied the application of porous materials for sound absorption. This review describes not only the process of sound absorption and the predictive behavioral models of porous materials that are characterized by their sound-absorption properties, but also analyses the development of the principles for designing foams and fibrous sound-absorbing materials, including their production. Some scenarios and perspectives on sound-absorbing structures and improvements to their properties are introduced in the conclusions of the paper. Gulia and Gupta [12] studied the damping of noise in a triple board for which they used a porous material and a sonic crystal with a specific resonant area. To suppress the sound transmission in the triple panel, they combined the characteristics of the material with porosity and a locally-resonant sonic crystal. An analytical method was used for this research. The results highlight two types of sudden decreases in the loss of sound transmission: Bragg’s dip and the dip caused by panel oscillation. To avoid a sudden drop in sound loss, the cavities of a porous material were filled with a medium. Yoon et al. [13] studied porous materials and optimized their topology to improve their noise absorption properties. The goal of their research was to develop a numerical method to appropriately distribute the solid elements in a layer of a porous structure with a constant thickness so that the noise would be completely absorbed. No special process was used for the initial distribution of the solid inclusions. Only then were the systematic multiple distributions of the solid inclusions implemented in a given porous layer, which was realized using a topology optimization formula. The results of the finite element-based numerical method showed that different types of resonances appear in the optimized layer, which absorbs noise for all considered frequencies simultaneously. The influence of processing conditions on the quality of the geometry of the lattice structures was assessed by Rosli et al. [14], whose research investigated the effects of the processing parameters of the fused deposition modelling (FDM) technique on the geometrical quality of an ABS polymer lattice structure. The variations in the layer thickness of the FDM machine used in this study were 70 μm, 200 μm, and 300 μm. Examinations of the diameter circularity of the printed lattice blocks were conducted via direct measurements and formulations from a previous study. It was found that a layer thickness of 200 μm produced a more accurate strut diameter with a reliable mechanical response. Kim et al. [15] developed experiments to investigate the semi-active control of a sophisticated porous material related to its ability to absorb noise. The authors developed a smart structure that can reduce sound in a wide range of frequencies when semi-active regulation is realized through the application of a magnetic reactive material. Jimbo and Tateno [16] focused on creating a lattice structure with isotropic tensile strength that can be produced in an additive way. ABS material and geometry with a body-centered cubic (BCC) structure were selected to be studied. This type of cell was proposed to eliminate the anisotropic qualities caused by additive manufacturing. The specimens were produced via FFM technology, and the hypothesis of the isotropic tensile strength properties of such a structure was confirmed by experimental tests. Salomons et al. [17] studied the operation of soundwaves for fluid flow in a structure using the Lattice Boltzmann method (LBM). In the published manuscript, this method was applied to the analysis of noise propagation in several different environments, such as a free field, porous and nonporous structures, the sound barrier, and a windy atmosphere. The results obtained using the LBM were compared with the corresponding results computed based on the acoustic equations. It was concluded that the LBM is an appropriate method for soundwave investigations, but that it is necessary to take into account that the dispersion of soundwaves within the LBM is usually much greater than their dissipation in the air. Zvolensky et al. [18] investigated the behavior of noise propagation through a porous material and the specific parameters of noise pollution. The authors focused on a computer simulation of the sound transmitted through wagon walls and on the usage of noise analyses during the operation of a train. In the article, the acoustic characteristics of the primary materials were compared with those of the new ones. Chen et al. [19] were involved in the development of an acoustic superlens based on single-phase lattice metamaterials in the shape of a star made of steel. The authors invented low-density superlens which can reach a sound focus over the diffraction limit. These lattice structures offer rich resonances to induce abnormal dispersion effects, as specified by the negative parameter indices. Their study showed that this type of structure has double negative index properties that can mediate these effects for sound in water.

The present work aims to investigate the different factors that affect the sound absorption properties of 3D-printed ABS materials with various open-pore structures. Although many studies have been published related to the sound properties of porous materials, the proposed types of structures (produced from an ABS material) have not yet been investigated.

## 2. Materials and Methods

### 2.1. Materials

The specimens for the experimental tests were produced from an ABS (acrylonitrile-butadiene-styrene) material (Smart Materials 3D, Alcala la Real, Spain), which is one of the most commonly-used thermoplastic polymers for 3D printing technology. Due to its physical properties, this material is very often chosen to produce a wide range of components for use in relatively safe machines that are easy to operate. It is resistant to abrasion and high temperatures (its melting point is 200 °C/392 °F), and it is lightweight [20].

Its mechanical properties, such as impact and tensile strength, as well as stiffness, are very good. ABS is soluble in acetone. However, ABS also resists many chemical formulas. Moreover, this plastic has good surface quality and flame retardancy. It is recyclable and available to both professionals and the general public. Consequently, ABS is an ideal material for the production of inexpensive prototypes and architectural models for engineers or research departments, as well as for the creation of inexpensive material handling equipment [21,22].

### 2.2. Samples Production

**Figure 1:**
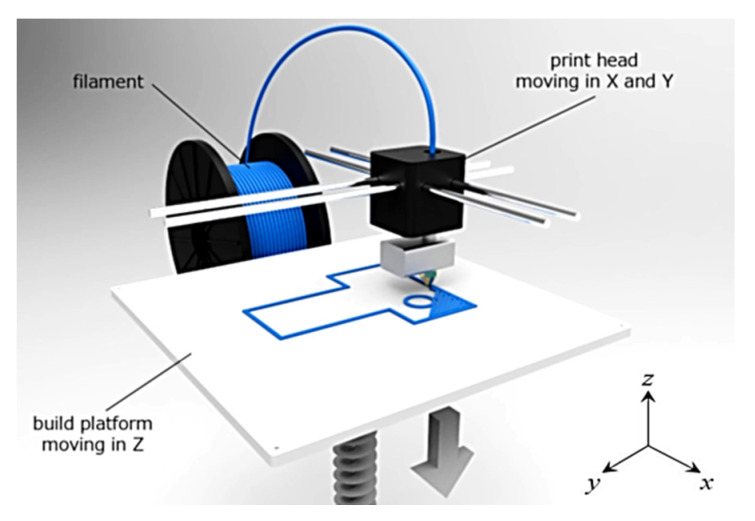
Principle of the Fused Deposition modelling (FDM) technique.

ABS is a material that is primarily used by 3D printers based on the FDM (Fused Deposition modelling) technique (see Figure 1).

**Figure 2:**
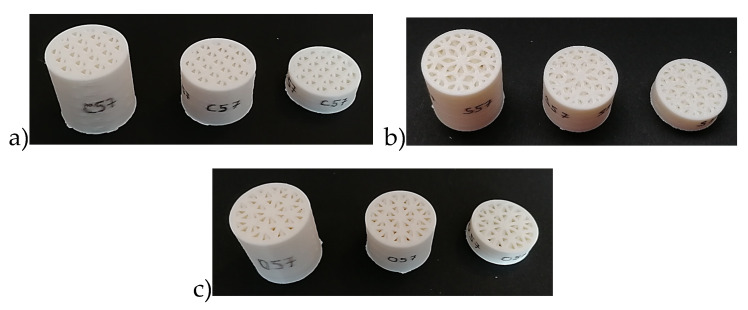
Tested samples: (**a**) Cartesian structure, (**b**) Starlit structure, and (**c**) Octagonal structure.

The material for 3D printing is prepared in the form of a long filament that is wound on a spool. This spool enters through the print head, which can move in the *x* and *y* axes (i.e., the plane of the printing bed). The movement of the head is digitally controlled based on the shape of the produced body in the given layer. The molten material is applied in a thin layer on the building platform to which it adheres. After the deposition of the first layer, the platform moves down, and a new layer is applied. This process repeats until the new component is finished. One of the advantages of 3D printing technology is the possibility to make parts with complex shapes that are non-manufacturable (or very difficult) with other methods. Such products include lightweight components with internal porous structures. The mechanical properties of porous structures are significantly affected by the material used and by the geometry of the structure. A very important parameter of porous materials is the “relative density”, or “Volume ratio *V_r_*”. This parameter expresses how much cell space is filled with material, and is described by the Equation (1) [23]:

(1) $$V_{r}\left(\%\right)=\frac{V_{S}}{V_{T}}\times 100$$

where *V_S_* is the volume of the solid phase and *V_T_* is the total body volume. In this study, three types of lattice structures (Cartesian, Starlit, and Octagonal) were modelled and produced from the plastic material ABSplus-P430 Ivory using the FDM technique. All the samples had a cylindrical shape with an outer diameter of φ29 mm (based on the testing device requirements) and three different lengths (thicknesses), i.e., 10, 20, and 30 mm. The core of every sample was filled with a lattice structure with a volume ratio *V_r_* = 57%, while the thickness of the outside cylindrical shell (fully filled by the material) was 2 mm. All basic cell types were modelled with the outer horizontal, outer vertical, and inner angular beams/struts. The *z* axis is the building axis, normal to the building platform (plane *xy*). The volume ratio was controlled by using a strut diameter of 1.4 mm. The structure types and their characteristics are presented in Table 1, while the produced samples are shown in Figure 2.

### 2.3. Measurement Methodology

#### 2.3.1. Sound Absorption Coefficient

**Figure 3:**
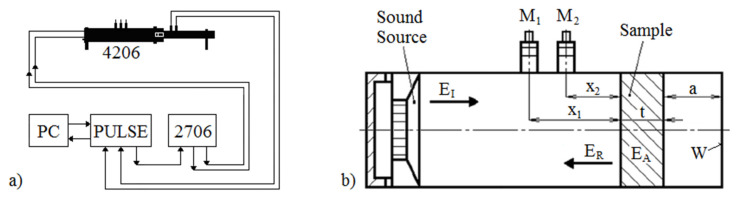
Schematic diagram of the apparatus for measuring the sound absorption coefficient (**a**) and a schematic of the impedance tube equipment (**b**). Legend of the abbreviations: *a*—air gap size; *E_A_*—absorbed acoustic energy; *E_I_*—incident acoustic energy; *E_R_*—reflected acoustic energy; M_1_, M_2_—measuring microphones; *t*—sample thickness; W—solid wall; *x*_1_, *x*_2_—microphone distances from the tested ABS sample.

If the incident acoustic energy *E_I_* is propagated from a noise source to a material surface, some of this incident energy is reflected from the surface. The remainder of the incident acoustic energy is absorbed by the tested acoustical material. The material’s ability to damp noise is expressed by the sound absorption coefficient *α*, which is defined by the following equation [24]:

(2) $$\alpha =\frac{E_{A}}{E_{I}}=1-\frac{E_{R}}{E_{I}}$$

where *E_A_* represents the absorbed acoustic energy and *E_R_* is the reflected acoustic energy (see Figure 3b). The basic function of sound-absorbing materials is to transform the incident acoustic energy into heat. There are two mechanisms by which the acoustic energy is dissipated: viscous-flow losses and internal friction [25].

#### 2.3.2. Noise Reduction Coefficient

The material’s ability to absorb sound depends on several factors, such as the material type, thickness, density, structure, excitation frequency, and temperature. The noise reduction coefficient (NRC) includes the effect of the excitation frequency on the sound absorption coefficient and is a single number, which is defined as the arithmetical average of the sound absorption coefficients at the frequencies 250, 500, 1000, and 2000 Hz [26,27]:

(3) $$NRC=\frac{\alpha_{250}+\alpha_{500}+\alpha_{1000}+\alpha_{2000}}{4}.$$

#### 2.3.3. Sound Absorption Properties

The sound absorption behavior of the investigated 3D-printed ABS materials was measured using a two-microphone acoustic impedance tube (BK 4206) in combination with a signal PULSE multi-analyzer (BK 3560-B-030) and a power amplifier (BK 2706) in the frequency range of 200–6400 Hz (Brüel & Kjær, Nærum, Denmark). A schematic diagram of the experimental apparatus is shown in Figure 3a. The normal incidence soundwave absorption of the tested samples of a given thickness *t* (from 10 to 30 mm) was experimentally determined for different air gap sizes *a* (ranging from 0 to 100 mm) behind the investigated ABS specimens, as shown in Figure 3b. All experiments were carried out at the ambient temperature of 23 °C.

The frequency dependencies of the sound absorption coefficient of the investigated materials were obtained by the transfer function method ISO 10534-2 [28], which is based on the partial standing wave principle. In this case, the normal incidence sound absorption coefficient *α* is expressed by the following formula [29,30]:

(4) $$\alpha =1-{\left|r\right|}^{2}=1-r_{r}^{2}-r_{i}^{2}$$

where *r* is the normal incidence reflection factor, and *r_r_* and *r_i_* are the real and imaginary components of the factor *r*, which is given by

(5) $$r=r_{r}+ir_{i}=\frac{H_{12}-H_{I}}{H_{R}-H_{12}}\cdot e^{2k_{0}\cdot x_{1}i}$$

where *H*_12_ is the complex acoustic transfer function, *H*_I_ is the transfer function for the incident wave, *H_R_* is the transfer function for the reflection wave, *k*_0_ is the wave number, and *x*_1_ is the distance between the investigated material sample and the microphone M_1_ (see Figure 3b). The transfer functions are expressed as follows:

(6) $$H_{12}=\frac{p_{2}}{p_{1}}=\frac{e^{k_{0}\cdot x_{2}i}+r\cdot e^{-k_{0}\cdot x_{2}i}}{e^{k_{0}\cdot x_{1}i}+r\cdot e^{\cdot k_{0}\cdot x_{1}i}}$$

(7) $$H_{I}=e^{-k_{0}\cdot \left(x_{1}\cdot x_{2}\right)i}$$

(8) $$H_{R}=e^{k_{0}\cdot \left(x_{1}\cdot x_{2}\right)i}$$

where *p*_1_ and *p*_2_ are the complex sound pressures at the two microphone positions, and *x*_2_ is the distance between the investigated material sample and the microphone M_2_ (see Figure 3b).

## 3. Results and Discussion

This section explores the different factors that influence the sound absorption properties of the investigated 3D-printed, open-pore ABS materials, whose designations are as follows: The sample designation is first described by its structure type (see Table 1). Subsequently, the sample designation consists of two numbers. The first number represents the sample thickness (in mm). The second number defines the air gap size *a* (in mm) behind the sample inside the impedance tube (see Figure 3b).

### 3.1. Frequency Dependencies of the Sound Absorption Coefficient

#### 3.1.1. Effect of Structure Type

**Figure 4:**
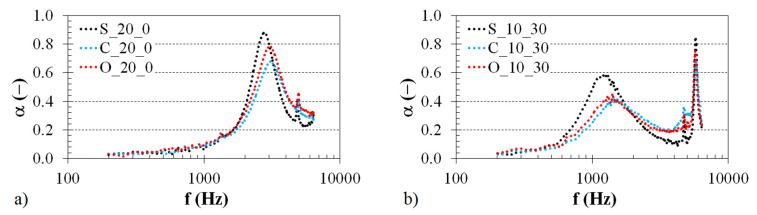
Effect of the 3D-printed ABS material structure on the frequency dependencies of the sound absorption coefficient; (**a**) sample thickness *t* = 20 mm, air gap size *a* = 0 mm, (**b**) sample thickness *t* = 10 mm, air gap size *a* = 30 mm.

The effect of the structure type on the sound absorption properties of the investigated 3D-printed ABS materials is demonstrated in Figure 4. Examples of the frequency dependencies of the sound absorption coefficient for the ABS samples of the same thickness (i.e., *t* = 20 mm) are shown in Figure 4a. In this case, the samples were mounted directly on the solid wall W (i.e., with *a* = 0 mm) inside the impedance tube (see Figure 3b). Similarly, the structural effect of the tested 3D-printed ABS materials (with *t* = 10 mm and *a* = 30 mm) is presented in Figure 4b. It is evident from these comparisons that the structural effect on sound absorption is negligible at low excitation frequencies, namely at *f* < 2 kHz (see Figure 4a) and at *f* < 700 Hz (see Figure 4b). Subsequently, better sound absorption properties were obtained for the ABS samples, which were produced with the Starlit structure. The ability of open-porous material structures to damp sound is related to the airflow resistivity of these structures. Generally, increasing the airflow resistivity improves sound absorption properties [31,32] in the whole frequency range but only up to an intermediate value. If the porous materials are too acoustically resistive and their airflow resistivity is very high, the sound absorption properties of these materials are very low during the propagation of an acoustic wave through their porous structures. The Starlit structure (see Table 1) is characterized by more complicated pore shapes compared to the Cartesian and Octagonal structures, resulting in higher airflow resistivity of the open-porous Starlit structure. For these reasons, the ABS samples produced with the Starlit structure exhibit better sound absorption performance than the other open-porous structures. This phenomenon is accompanied by higher internal friction during the propagation of an acoustic wave through this structure, and by greater transformation of incident acoustic energy into heat. This effect was observed in the frequency ranges of 2–3 kHz (see Figure 4a) and 0.7–1.9 kHz (see Figure 4b). Conversely, the 3D-printed ABS samples produced with the Cartesian and Octagonal structures, whose frequency dependencies of sound absorption are very similar, are more suitable for damping sound at higher excitation frequencies, namely in the frequency ranges *f* = 〈3.0; 6.4〉 kHz (see Figure 4a) and *f* = 〈1.9; 6.4〉 kHz (see Figure 4b). Similar structural effect results were found for the 3D-printed ABS samples further tested independently of the sample thickness and the air gap size.

#### 3.1.2. Effect of Material Thickness

**Figure 5:**
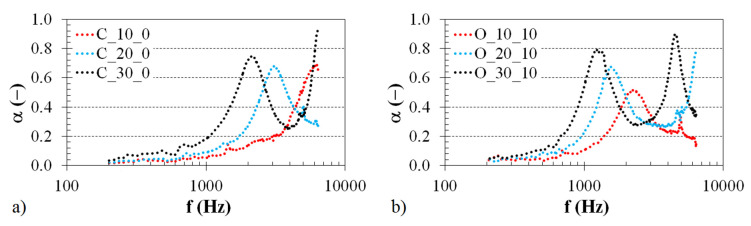
Effect of the sample thickness on the frequency dependencies of the sound absorption coefficient for the investigated ABS samples with a (**a**) Cartesian and (**b**) Octagonal structures.

A material’s ability to reduce noise is significantly influenced by its thickness. Figure 5 demonstrates the effect of varying the sample thickness when the tested sample is mounted directly (see Figure 5a) on a solid wall or placed at a distance of 10 mm from the wall (see Figure 5b). The sample thickness significantly improves sound absorption towards lower excitation frequencies. However, increasing the sample thickness leads to higher production costs for the 3D-printed ABS samples. The production costs of the 3D printing of components are generally influenced by various factors, such as the type of 3D-printed material, the printing layer thickness, the printing speed, the dimensions of the 3D-printed components, and the printing time. Under the same 3D printing process parameters of a given structure type, the production costs increase by increasing printing time. Because the ground plan dimensions of the investigated open-porous ABS samples are identical, an increase in sample thickness (or sample volume) increases the printing time and thus the production costs of the 3D printing process [33,34]. Therefore, this method is not effective in terms of noise reduction.

#### 3.1.3. Effect of the Air Gap Size

**Figure 6:**
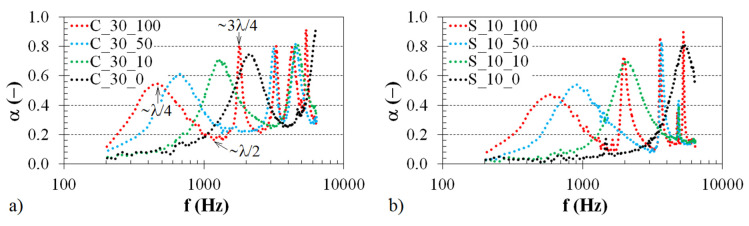
Effect of the air gap size on the frequency dependencies of the sound absorption coefficient for the investigated ABS samples with a (**a**) Cartesian and (**b**) Starlit structure.

The air gap size *a* between the tested ABS sample and the solid wall also has a significant effect on sound damping. As shown in Figure 6, it is possible to observe a certain number of maxima and minima of the sound absorption coefficient over the whole frequency range depending on the sample type. These effects are explained by the sound reflections from the solid wall inside the impedance tube and by the wavelength of sound *λ*, which is defined as the speed of sound divided by the frequency [35].

At the wall surface, the acoustic pressure reaches its maximum value, but the air particle velocity is zero. Conversely, in the case of a quarter wavelength (see Figure 6a) from the wall W (see Figure 3b), the acoustic pressure is zero, and the air particle velocity is maximum. When the investigated porous material is placed at a quarter-wavelength distance from the solid wall, it is possible to obtain the maximum sound absorption because the air particle velocity is maximum. Similarly, at a half-wavelength, the air particle velocity is minimum, and the sound absorption coefficient is also minimum [35,36]. For these reasons, sound absorption maxima occur at odd multiples of quarter wavelengths in the standing-wave antinodes at the excitation frequencies:

(9) $$f=\frac{c\cdot \left(2n+1\right)}{4l}$$

where *c* is the speed of sound, *n* is an integer (*n* = 0, 1, 2…), and *l* is the sample distance from the solid wall (*l* = *a* + *t*/2). Similarly, the sound absorption minima are obtained at even multiples of quarter wavelengths in standing-wave nodes at the excitation frequencies:

(10) $$f=\frac{c\cdot n}{2l}$$

Table 2 presents the values of the primary sound absorption maxima *α_max_*_1_ (corresponding to a quarter-wavelength, i.e., *λ*/4), the primary sound absorption minima *α_min_*_1_ (corresponding to a half-wavelength, i.e., *λ*/2), and the corresponding excitation frequencies *f_max_*_1_ and *f_min_*_1_ of the ABS samples whose frequency dependencies of the sound absorption coefficient are depicted in Figure 6.

Here, the frequencies *f_max_*_1_ and *f_min_*_1_ generally decrease with an increase in the air gap size *a*. Therefore, it can be concluded that open-porous structures with air gaps are effective for improving sound absorption properties at low excitation frequencies instead of increasing the thickness of the sound absorber, which requires more materials [37].

#### 3.1.4. Effect of Excitation Frequency

The excitation frequency *f* is another important factor that influences the sound absorption properties of the investigated 3D-printed ABS samples. It is evident (see Figure 4, Figure 5 and Figure 6) that the highest value of the sound absorption coefficient is obtained at a given frequency depending on the sample type. For example, in the case of the sample with the Starlit structure that was mounted directly on the solid wall (i.e., without the air gap size) inside the impedance tube, the maximum value of the sound absorption coefficient *α_max_* = 0.81 was observed at the frequency *f* = 5328 Hz (see Figure 6b) for a sample thickness *t* = 10 mm. The same sample type (with a thickness *t* = 20 mm) that was directly mounted on the solid wall had the best sound absorption properties (*α_max_* = 0.88) at the frequency *f* = 2760 Hz (see Figure 4a). Similarly, the sample with a thickness *t* = 10 mm and the Starlit structure placed at a distance of 50 mm from the solid wall had the greatest ability to absorb sound (*α_max_* = 0.84) at the frequency *f* = 3648 Hz (see Figure 6b).

Generally, a very low material ability to absorb sound was observed at low excitation frequencies. In these cases, it is possible to increase the sample thickness (see Figure 5) to improve the sound damping of the open-porous materials. This method of noise elimination is not effective because the increase in material thickness is connected with the higher production costs of the investigated 3D-printed ABS materials. It is also possible to improve the sound absorption properties of the open-porous materials at low excitation frequencies via air-gap magnification (see Figure 6) behind the tested material sample.

The influence of the excitation frequency on sound absorption is considered based on the noise reduction coefficient defined according to Equation (3). The different factors that influence the noise reduction coefficient of the tested open-porous ABS samples are evaluated in the following section.

### 3.2. Noise Reduction Coefficient

As mentioned above, the noise reduction coefficient NRC is used to describe the average sound absorption performance of a given soundproofing material.

**Figure 7:**
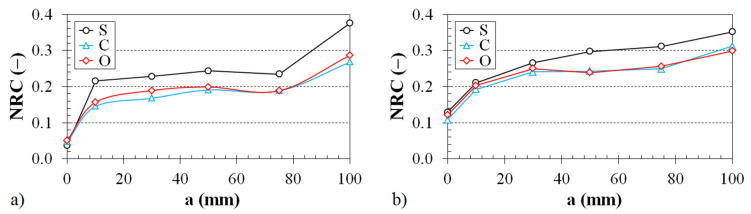
Effect of material structure on the noise reduction coefficient vs. air gap size dependencies for the investigated ABS samples with the following thicknesses: (**a**) 10 mm and (**b**) 20 mm.

**Figure 8:**
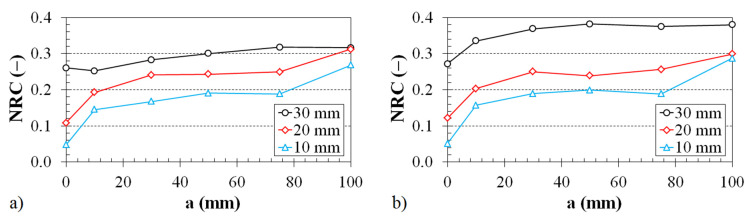
Noise reduction coefficient vs. air gap size dependencies for the investigated ABS samples with (**a**) Cartesian and (**b**) Octagonal structures. Inset legend: sample thickness.

The dependence of the noise reduction coefficient on the air space size behind the 3D-printed ABS samples inside the impedance tube (see Figure 3b) is demonstrated in Figure 7 and Figure 8.

The effect of the ABS sample structure on the noise reduction coefficient vs. the air gap size for the two various sample thicknesses is shown in Figure 7. The graphs in Figure 7 clearly show that the samples produced with the Starlit structure had higher noise reduction coefficient values, and thus, better sound absorption properties than the Cartesian and Octagonal structures. For example, for the ABS sample with a thickness *t* = 10 mm placed at a distance of 75 mm from the solid wall, a noise reduction coefficient NRC = 0.235 was observed for the ABS sample produced with the Starlit structure. However, when the ABS sample was produced with a Cartesian or Octagonal structure, the value of the noise coefficient (NRC ≅ 0.190) was lower (see Figure 7a). Similarly, for the ABS sample with a thickness of 20 mm that was placed at a distance of 100 mm from the solid wall, a higher noise reduction coefficient value was observed for the ABS sample produced with the Starlit structure (NRC = 0.352) compared to the ABS samples produced with Cartesian (NRC = 0.312) and Octagonal (NRC = 0.299) structures (see Figure 7b). It was found in this study that the lowest value of the noise reduction coefficient (NRC = 0.047) was observed for the ABS sample with a thickness *t* = 10 mm that was produced with a Cartesian lattice structure and was mounted directly on the solid wall (i.e., without the air gap size) inside the impedance tube. Conversely, the maximum value of the noise reduction coefficient (NRC = 0.406) was observed for the ABS sample of a thickness *t* = 30 mm, which was produced with the Starlit lattice structure and placed at a distance of 100 mm from the solid wall of the impedance tube. The above phenomenon is caused by the higher internal friction during the propagation of an acoustic wave through the Starlit structure, which leads to a higher transformation of the incident acoustic energy into heat during sound propagation through this structure compared to the Cartesian and Octagonal structures, whose noise reduction is very similar (see Figure 7). It was also found that the noise reduction coefficient generally increases with an increase in the material thickness *t* and the air gap size *a*. For example (see Figure 7a,b), if the ABS sample produced with the Octagonal structure is placed at a distance of 30 mm from the solid wall, the noise reduction coefficient increases from 0.189 (for *t* = 10 mm) to 0.250 (for *t* = 20 mm). For the ABS sample of thickness *t* = 10 mm with a Cartesian structure, the noise reduction coefficient increased (see Figure 7a) from 0.047 (for *a* = 0 mm) to 0.268 (for *a* = 100 mm). Likewise (see Figure 8a), the minimum value of the noise reduction coefficient (NRC = 0.108) for the ABS sample of thickness *t* = 20 mm with a Cartesian lattice structure was observed in such a case when the tested sample was mounted directly (i.e., with *a* = 0 mm) on the solid wall. When the same ABS sample was placed at a distance of 100 mm from the wall, the maximum value of the noise reduction coefficient (NRC = 0.312) was found. If this ABS sample type was mounted directly on the wall, the noise reduction coefficient increased from 0.047 (for *t* = 10 mm) to 0.260 (for *t* = 30 mm). Similarly, if this sample was placed at a distance of 100 mm from the wall, the noise reduction coefficient increased from 0.268 (for *t* = 10 mm) to 0.312 (for *t* = 30 mm). Similar results were observed for the ABS sample produced with the Octagonal lattice structure (see Figure 8b). For the ABS specimen with a thickness *t* = 20 mm, the noise reduction coefficient increases from 0.121 (for *a* = 0 mm) to 0.299 (for *a* = 100 mm). When this specimen is mounted directly on the solid wall, the noise reduction coefficient rises from 0.051 (for *t* = 10 mm) to 0.271 (for *t* = 30 mm). In the case of the maximum air gap size (i.e., with *a* = 100 mm) inside the impedance tube (see Figure 8b), the noise reduction coefficient of this ABS specimen increased from 0.287 (for *t* = 10 mm) to 0.379 (for *t* = 30 mm).

The effect of the ABS sample thickness on the noise reduction coefficient vs. the air gap size for two different types of 3D-printed structures (Cartesian and Octagonal) is presented in Figure 8. It is evident from Figure 8 that the noise reduction coefficient increases by increasing the sample thickness independently of the structure type and the air gap size. For this reason, sample thickness has a positive influence on sound absorption. It is also obvious that the noise coefficient generally increases with an increase in the air gap size.

**Figure 9:**
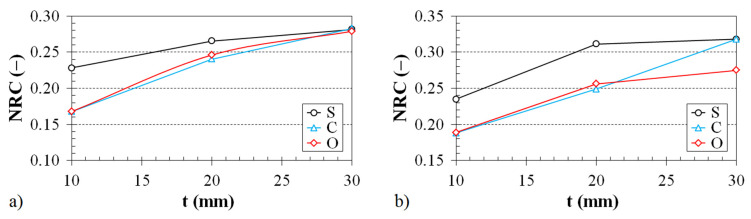
Effect of the material structure on the noise reduction coefficient vs. the material thickness dependencies for the investigated ABS samples placed at a distance from the wall inside the impedance tube: (**a**) 30 mm and (**b**) 75 mm.

**Figure 10:**
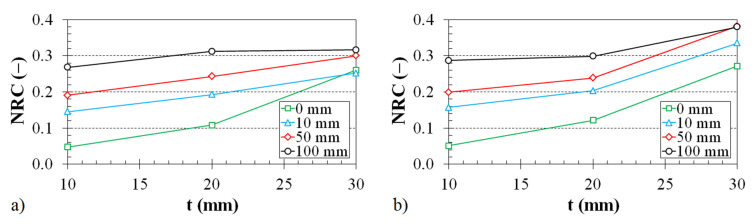
Noise reduction coefficient vs. material thickness dependencies for the investigated ABS samples with (**a**) Cartesian and (**b**) Octagonal structures. Inset legend: air gap size.

The dependence of the noise reduction coefficient on the thickness of the 3D-printed ABS samples is shown in Figure 9 and Figure 10.

The effect of the ABS sample structure on the noise reduction coefficient vs. the material thickness for two various air gap sizes behind the tested ABS samples inside the impedance tube is shown in Figure 9. It was found again (as with the effect of the air gap size on the noise reduction coefficient; see Figure 7) that the samples produced with the Starlit structure have better sound absorption properties than those with Cartesian and Octagonal structures. This effect is especially significant for smaller sample thicknesses (i.e., for *t* < 20 mm). It was also found that the noise reduction coefficient generally increases with an increase in the sample thickness and air gap size (see Figure 9).

The effect of the air gap size on the noise reduction coefficient vs. the material thickness for two different types of 3D-printed structures is demonstrated in Figure 10. The noise reduction coefficient increases with an increase in the air gap size and the sample thickness independently of the structure type. Therefore, the sound absorption properties of the investigated open-porous, 3D-printed ABS samples generally increase with an increase in the air gap size and material thickness.

## 4. Conclusions

In this work, we investigated the sound absorption properties of open-porous, 3D-printed ABS samples manufactured using Cartesian, Starlit, and Octagonal structures with the same volume ratios. It was found that the ABS samples produced with the Starlit structure exhibited a greater ability to damp noise compared to the other ABS structures examined.

Depending on the ABS sample thickness and the air gap size behind the tested samples inside the impedance tube, the noise reduction coefficient of the samples produced with Starlit structures was higher (the increment was between 0 and 0.1) than those with Cartesian and Octagonal structures. This result is related to the pore spaces and sizes, which have a notable effect on airflow resistivity and thus on the sound absorption performance of the investigated open-porous structures.

It was also found that low sound damping properties are generally observable at the low excitation frequencies of an acoustic wave. Hence, it was necessary to increase the material thickness, which was reflected by the improved sound absorption in the low-frequency region. However, increasing the sample thickness results in higher manufacturing costs for the 3D-printed ABS samples. Therefore, this method is not effective for noise reduction. For this reason, we recommend applying open-porous sound absorbers with air gaps to improve sound damping at low excitation frequencies. This effect was particularly significant when increasing the air gap size, which resulted in a shift of the primary sound absorption maxima to lower excitation frequencies. Depending on the ABS sample thickness and structure type, the frequency of the primary sound absorption maxima decreased from *f _max_*_1_ ≅ 〈2.0; 6.1〉 kHz for the ABS samples mounted directly (without an air gap) on the solid wall of the impedance tube to *f_max_*_1_ = 〈416; 656〉 Hz for the ABS samples placed at a distance of 100 mm from the wall.

## Figures and Tables

**Table 1:** Types of 3D-printed structures.

Structure Type	Volume ratio (%)	Label	Front View	Strut Diameter	Cell Sizes (mm)
Cartesian	57	C_57	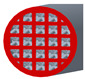	1.4	x = 5
					y = 5
					z = 5
Starlit	57	S_57	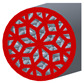	1.4	x = 9
					y = 9
					z = 5
Octagonal	57	O_57	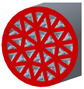	1.4	x = 7
					y = 7
					z = 5

**Table 2:** Primary sound absorption maxima and minima and their corresponding frequencies depending on the air gap size for the investigated ABS samples with Cartesian and Starlit structures.

Structure Type	*t* (mm)	*a* (mm)	*f_max_*_1_ (Hz)	*α_max_*_1_ (−)	*f_min_*_1_ (Hz)	*α_min_*_1_ (−)
Cartesian	30	0	2096	0.76	4184	0.25
		10	1304	0.73	3000	0.23
		50	656	0.63	1864	0.22
		100	432	0.55	1136	0.17
Starlit	10	0	5328	0.81	-	-
		10	2064	0.71	5912	0.15
		50	904	0.55	2992	0.08
		100	552	0.48	1312	0.09
